# Changes in key vaginal bacteria among postpartum African women initiating intramuscular depot-medroxyprogesterone acetate

**DOI:** 10.1371/journal.pone.0229586

**Published:** 2020-03-05

**Authors:** Bridget M. Whitney, Brandon L. Guthrie, Sujatha Srinivasan, Kenneth Tapia, Eric Munene Muriuki, Bhavna H. Chohan, Jacqueline M. Wallis, Congzhou Liu, R. Scott McClelland, David N. Fredricks, Alison C. Roxby

**Affiliations:** 1 Department of Epidemiology, University of Washington, Seattle, WA, United States of America; 2 Department of Global Health, University of Washington, Seattle, WA, United States of America; 3 Vaccine and Infectious Disease Division, Fred Hutchinson Cancer Research Center, Seattle, WA, United States of America; 4 Institute of Infectious and Tropical Diseases, University of Nairobi, Nairobi, Kenya; 5 Department of Medicine, University of Washington, Seattle, WA, United States of America; Centre Pasteur du Cameroun, CAMEROON

## Abstract

**Background:**

The ECHO trial has relieved apprehension about intramuscular depot medroxyprogesterone acetate (DMPA-IM), however it is still important to understand how DMPA-IM affects the vaginal environment. We sought to describe how DMPA-IM initiation influences vaginal bacteria associated with HIV acquisition in postpartum women.

**Methods:**

Vaginal swabs were collected for Nugent score determination and taxon-specific quantitative PCR of eight bacteria. Enrollment occurred at contraceptive initiation (DMPA-IM or non-hormonal contraception (non-HC)) and repeat vaginal swabs were collected after three months. Generalized estimating equations were used to estimate changes in Nugent score, total bacterial load, and taxa concentrations among contraceptive groups.

**Results:**

Women who chose DMPA-IM (n = 33) were more likely to be married (97%vs.67%) and have resumed intercourse since delivery (52%vs.29%) compared to women who chose non-HC (n = 21). After three months, significant decreases in the concentrations of *Sneathia* species, *Mycoplasma hominis*, and *Parvimonas* species Type 1 were seen among non-HC users, however concentrations remained stable among DMPA-IM users; contraceptive method was associated with significantly different changes in *M*. *hominis* concentration between groups (p = 0.010).

**Conclusions:**

Our findings suggest that postpartum use of DMPA-IM and non-HC may have differential impacts on the vaginal concentrations of some bacteria that have previously been associated with HIV acquisition.

## Introduction

The vaginal microbiome plays a key role in women's reproductive health. Hydrogen peroxide-producing *Lactobacillus*-dominant vaginal bacterial communities are considered optimal for health [1, 2]. Communities dominated by anaerobic bacteria, or non-optimal microbiota, as well as specific microbial taxa, are associated with subclinical inflammation, poor reproductive health outcomes, and sexually transmitted infections [3–6]. Hormones, including estrogens and progestogens, play important roles in vaginal microbial ecology [7–9], and exogenous hormones, such as contraceptives, may induce important changes in the composition of vaginal microbiota and production of soluble factors by bacteria.

Injectable progestin-only contraceptives, including intramuscular depot-medroxyprogesterone acetate (DMPA-IM), are the most commonly used contraceptives among women in sub-Saharan Africa [10], where the burden of vaginal dysbiosis is highest [11] and 25.7 million people live with HIV [12]. DMPA-IM inhibits the secretion of pituitary gonadotropins, resulting in anovulation and decreased production of estrogen [13]. Reduced levels of estrogen have been associated with glycogen suppression [8, 14], and glycogen is an important substrate for *Lactobacillus* species [14–16]. Consequently, a reduction of glycogen in the female reproductive tract could shift the vaginal microbiome to an anaerobic, non-optimal state. Changes in vaginal bleeding patterns and perturbation of the regular menstrual cycle associated with DMPA-IM initiation may also impact the vaginal microbiome [9, 17]. Additionally, previous observational research has suggested that DMPA-IM use may be associated with increased risk of HIV acquisition [18–21]. While recently released findings from The Evidence for Contraceptive Options and HIV Outcomes (ECHO) Trial have generally reduced clinical concern about DMPA-IM use in women at risk of HIV [22], there is still uncertainty in the field as to whether DMPA-IM increases the risk of HIV acquisition relative to non-HC methods [23]. Therefore, it is important to understand how DMPA-IM affects the vaginal environment, including vaginal microbiota.

Several anaerobic bacteria have been identified as significantly associated with increased HIV acquisition, including: *Prevotella* species (*P*. *melaninogenica* [5], *P*. *bivia* [5]), *Mycoplasma* species (*Mycoplasma* spp. [5], *M*. *hominis* [6]), *Sneathia* species (*S*. *sanguinegens* [5], *Leptotrichia/Sneathia* (now *Sneathia* spp.) [6]), *Parvimonas* species type 2 [6], *Gemella asaccharolytica* [6], *Eggerthella* species Type 1 [6], and *Megasphaera* species (*Megasphaera* spp. Types 1 and 2 (combined assay) [6]). Observed heterogeneity in species-level determination of *Mycoplasma* and *Sneathia* species between studies may be due, at least in part, to different laboratory and bioinformatics methods [24]. In addition, unpublished data from two studies have also linked *P*. *bivia* with increased HIV acquisition risk [25, 26], and one of the studies reported associations between *M*. *hominis*, *Leptotrichia/Sneathia* (now *Sneathia* spp.), *Parvimonas* sp. type 2, *G*. *asaccharolytica*, *Eggerthella* sp. Type 1, and *Megasphaera* sp. Type 1 and increased HIV acquisition risk [26], replicating the published findings from other East African cohorts [6].

A limited number of studies have evaluated DMPA-IM’s effect on the overall vaginal microbiome [27–32], and to our knowledge there are no published studies specifically evaluating the effect of DMPA-IM on the concentration of a majority of bacterial taxa associated with HIV acquisition. Additionally, there is a gap in this data for postpartum women. Therefore, we sought to investigate if DMPA-IM use is associated with increases in bacteria previously associated with HIV acquisition [5, 6, 25, 26]. Based on pilot data from a cohort of Kenyan women [31], we hypothesized that quantities of *G*. *vaginalis* would decline in DMPA-IM users and other anaerobic bacteria would increase to fill the opening.

## Materials and methods

### Study setting, subjects, and design

We designed a prospective cohort study of postpartum women to assess how DMPA-IM affects vaginal environment, including alterations to vaginal microbiota, in the three months following DMPA-IM initiation. Breastfeeding women, 6–14 weeks postpartum who sought contraception counseling at a public primary care clinic in Nairobi, Kenya were recruited for enrollment. Because the menstrual cycle is associated with changes in vaginal microbiota [9, 17, 33], enrollment was limited to lactating, amenorrheic women to reduce expected variability at baseline. Women were eligible for enrollment if they were HIV-negative and chose DMPA-IM or non-HC (condoms, lactational amenorrhea, rhythm) as their contraceptive method. Women already using a hormonal contraceptive or unwilling to learn their HIV status were ineligible. Women with evidence of cervicitis or STI at enrollment were excluded.

Women attended two or three study visits depending on contraceptive method. At the enrollment visit women chose their contraceptive method; those who chose DMPA-IM received an injection from study staff, verifying the exact time of DMPA-IM administration. DMPA-IM users were asked to return approximately 9–14 days later for a brief visit coinciding with typical peak plasma medroxyprogesterone acetate (MPA) concentrations, the active component of DMPA-IM [34, 35]. All women were asked to return for a follow-up visit 3 months post-enrollment. Demographic, health, and sexual activity information were collected via questionnaires at each visit. Vaginal swabs for microbiota evaluation were collected during pelvic exams; swabs were taken bilaterally in the space that includes right and left lateral fornix and the distal two-thirds of the vaginal wall. At the enrollment visit, all specimens were collected before DMPA-IM administration. Swabs were not collected from women with vaginal bleeding, including any spotting related to DMPA-IM initiation. If women had any vaginal bleeding or spotting, they were asked to return for swab collection when bleeding had stopped.

Vaginal swabs were cryopreserved at –80°C and batch shipped on dry ice to the Fred Hutchinson Cancer Research Center (Seattle, WA, USA) for analysis.

### Laboratory procedures

Peripheral blood was used for point-of-care HIV testing (Determine rapid enzyme-linked immunosorbent assay, Abbott, Abbott Park, Illinois). Vaginal saline wet mounts were examined microscopically for the presence of motile trichomonads and fungal elements. Gram stains of vaginal fluid were used for evaluation of bacterial vaginosis (BV), with Nugent scores of ≥7 considered a diagnosis of Nugent-BV (method of Nugent and Hillier [36]).

Quantitative PCR (qPCR) was performed for eight bacterial taxa (*Gardnerella vaginalis*, *M*. *hominis*, *Sneathia* species, *G*. *asaccharolytica*, *Eggerthella* sp. Type 1, *Megasphaera* spp. Types 1 and 2 (combined assay), *Parvimonas* sp. Type 1, and *Parvimonas* sp. Type 2), using previously described methods [6, 37, 38]. Detailed methodology on DNA extraction and amplification have been detailed previously [6, 39–41] and are described in S1 File.

### Statistical analyses

Bacterial DNA concentrations were log_10_-transformed to normalize their distribution. DNA concentrations, when not detected, were assigned a value of half the lower limit of detection (LLD) of the assay (1.495 log_10_ copies/swab for all taxa except *Eggerthella* sp. Type 1 which had a LLD of 1.796 log_10_ copies/swab).

Our primary analysis was evaluation of changes in Nugent score, total bacterial load, and the concentration of the eight selected bacterial taxa among DMPA-IM users and non-HC users between enrollment and follow-up, as well as to compare changes over time between the contraceptive groups. Change from enrollment to the three-month follow-up visit among all women was estimated separately for each outcome using generalized estimating equations (GEE) with an interaction term between contraceptive group and time (days from enrollment/DMPA-IM injection to swab collection). Models including all women were adjusted for time from delivery to enrollment, as well as important potential confounders selected *a priori*, including age, marital status, and resumption of intercourse at enrollment. Patterns of change over time were visualized using Spaghetti plots, with trend lines for the mean change in the outcomes for each contraceptive group estimated from the GEE models.

Due to a large proportion of women having undetectable concentrations of the eight selected bacterial taxa, we also examined the pattern of taxon detection over follow-up and evaluated change in detectability of each taxa by contraceptive group using McNemar’s test. Sensitivity analyses were performed on the subsets of participants with at least one detectable value for that taxon during follow-up (women with a value above the lower limit of detection for a specific taxon at one or both visits). In these subsets, we only adjusted for time from delivery to enrollment since a majority of the taxa were detectable in 20 women or less and we did not want to overfit the models [42]. Patterns of change were visualized using Spaghetti plots, with trend lines for mean change among women with at least one detectable value for that taxon estimated from the GEE models.

Within the DMPA-IM group, we assessed whether the time of peak serum MPA concentrations was associated with changes in the outcomes using similar methods as above. We evaluated patterns of change over follow-up, including change from enrollment to the two-week post-injection visit, when MPA serum concentrations are approximately at peak by published pharmacokinetic data [34, 35], and from the two-week post-injection visit to the three-month follow-up visit.

For all associations, the significance level was set at p<0.05. Analyses were performed using Stata version 14 (StataCorp, College Station TX). The research protocol was approved by the Kenyatta National Hospital Ethics and Research Committee and the University of Washington Institutional Review Board. Written informed consent was obtained in English or Kiswahili from all participants.

## Results

### Participant characteristics

We enrolled 54 women, 33 (61%) of whom chose to initiate DMPA-IM. Women who chose DMPA-IM sought contraception counseling sooner than women who chose non-HC methods (7.1 weeks (standard deviation (SD): 2.0) post-delivery vs. 9.9 weeks (SD: 3.5) post-delivery) (Table 1). A higher proportion of DMPA-IM users were married (97% vs. 67%) and had resumed sexual intercourse by enrollment (52% vs. 29%) (Table 2). There was a non-significant trend towards increased Nugent-BV at baseline among women who chose DMPA-IM compared to those who chose non-HC (unadjusted Odds Ratio (OR) = 2.71, 95% Confidence Interval (CI): 0.83–8.87); this trend was not present at follow-up. Vaginal washing was common (DMPA-IM: 52%, non-HC: 62%) and became more prevalent in both groups, with a larger increase among DMPA-IM users (DMPA-IM: 85%, non-HC: 72%). Of the enrolled women, 44 (81%) women returned for their three-month follow-up visit. Time between the enrollment visit and the three-month follow-up visit was comparable between the two contraceptive groups (84 days for non-HC users [interquartile range (IRQ): 84–90 days] and 84 days for DMPA-IM users [IRQ: 84–89 days]). There were no incident HIV infections during follow-up.

**Table 1:** Participant sociodemographic characteristics by contraceptive group (N = 54).

Characteristic^a^	Non-HC (n = 21)	DMPA-IM (n = 33)
Age	26.0 (6.0)	23.1 (3.1)
Married	14 (66.7%)	32 (97.0%)
Education level at enrollment		
Primary school or less	9 (42.9%)	18 (54.6%)
Some secondary school/Completed Secondary	10 (47.6%)	10 (30.3%)
Some college/Higher education	2 (9.5%)	5 (15.2%)
Number of household residents	4.8 (1.6)	3.7 (1.1)
Median monthly household income in USD (IQR)^b^	98 (73–147)	98 (29–196)
Age at first intercourse	18.5 (2.1)	18.4 (2.3)
Lifetime number of sex partners	1.7 (1.0)	2.0 (1.1)
Number of pregnancies	2.1 (1.4)	1.8 (1.0)
Vaginal delivery (most recent delivery)	18 (85.7%)	28 (84.8%)
Time since most recent delivery (weeks)	9.9 (3.5)	7.1 (2.0)
Previous use of any family planning method	10 (47.6%)	11 (33.3%)

Abbreviations: DMPA-IM, intramuscular depot-medroxyprogesterone acetate; HC, hormonal contraception; IQR, interquartile range; SD, standard deviation.

^a^ Data presented as mean (SD) unless otherwise noted.

^b^ Non-HC users: n = 16; DMPA-IM users: n = 19.

**Table 2:** Key participant characteristics by contraceptive group at enrollment and the three-month follow-up visit.

	Enrollment (N = 54)		Follow-up (N = 44)	
Characteristic^a^	Non-HC (n = 21)	DMPA-IM (n = 33)	Non-HC (n = 18)	DMPA-IM (n = 26)
Current breastfeeding	21 (100%)	33 (100%)	18 (100%)	26 (100%)
Exclusive breastfeeding	20 (95.2%)	31 (93.9%)	13 (72.2%)	19 (73.1%)
Mean vaginal pH^b^ (SD)	5.3 (0.9)	5.5 (0.6)	4.9 (0.8)	5.2 (0.8)
BV (Nugent score ≥7)	7 (33.3%)	19 (57.6%)	5 (27.8%)	8 (30.8%)
Mean Nugent score (SD)	3.6 (3.7)	5.3 (3.8)	2.7 (3.7)	3.3 (3.7)
Self-reported abnormal vaginal discharge^1^	3 (14.3%)	0	0	2 (7.7%)
Clinician-reported abnormal vaginal discharge^c^	0	2 (6.1%)	0	1(3.9%)
Abnormal vaginal itching^1^^,^^d^	5 (23.8%)	0	1 (5.6%)	3 (11.5%)
Vaginal washing^1^	13 (61.9%)	17 (51.5%)	13 (72.2%)	22 (84.6%)
Vaginal bleeding^1^	3 (14.3%)	9 (27.3%)	7 (38.9%)	17 (65.4%)
Use of antibiotics or metronidazole^2^	0	1 (3.0%)	1 (5.6%)	2 (7.7%)
Resumed sexual intercourse since delivery (enrollment) or recent sex^1^ (follow-up)	6 (28.6%)	17 (51.5%)	10 (55.6%)	18 (69.2%)
Currently using condoms^3^^,^^d^	1 (16.7%)	0	8 (80.0%)	2 (11.1%)

Abbreviations: BV, bacterial vaginosis; DMPA-IM, intramuscular depot-medroxyprogesterone acetate; HC, hormonal contraception; SD, standard deviation.

Enrollment: visit DMPA-IM was administered; Follow-up: visit three-months post DMPA-IM initiation.

^a^ Data presented as n (%) unless otherwise noted.

^b^ Follow-up Non-HC users: n = 17; Follow-up DMPA-IM users: n = 23.

^c^ Enrollment Non-HC users: n = 20; Baseline DMPA-IM users: n = 32; Follow-up Non-HC users: n = 17; Follow-up DMPA-IM users: n = 23.

^d^ Enrollment Non-HC users: n = 20; ^d^ Follow-up DMPA-IM users: n = 17.

^1^ Past week.

^2^ Past month.

^3^ Percentage among of those reporting recent sex.

### Detection of the eight taxa assessed among participants

**Fig 1:**
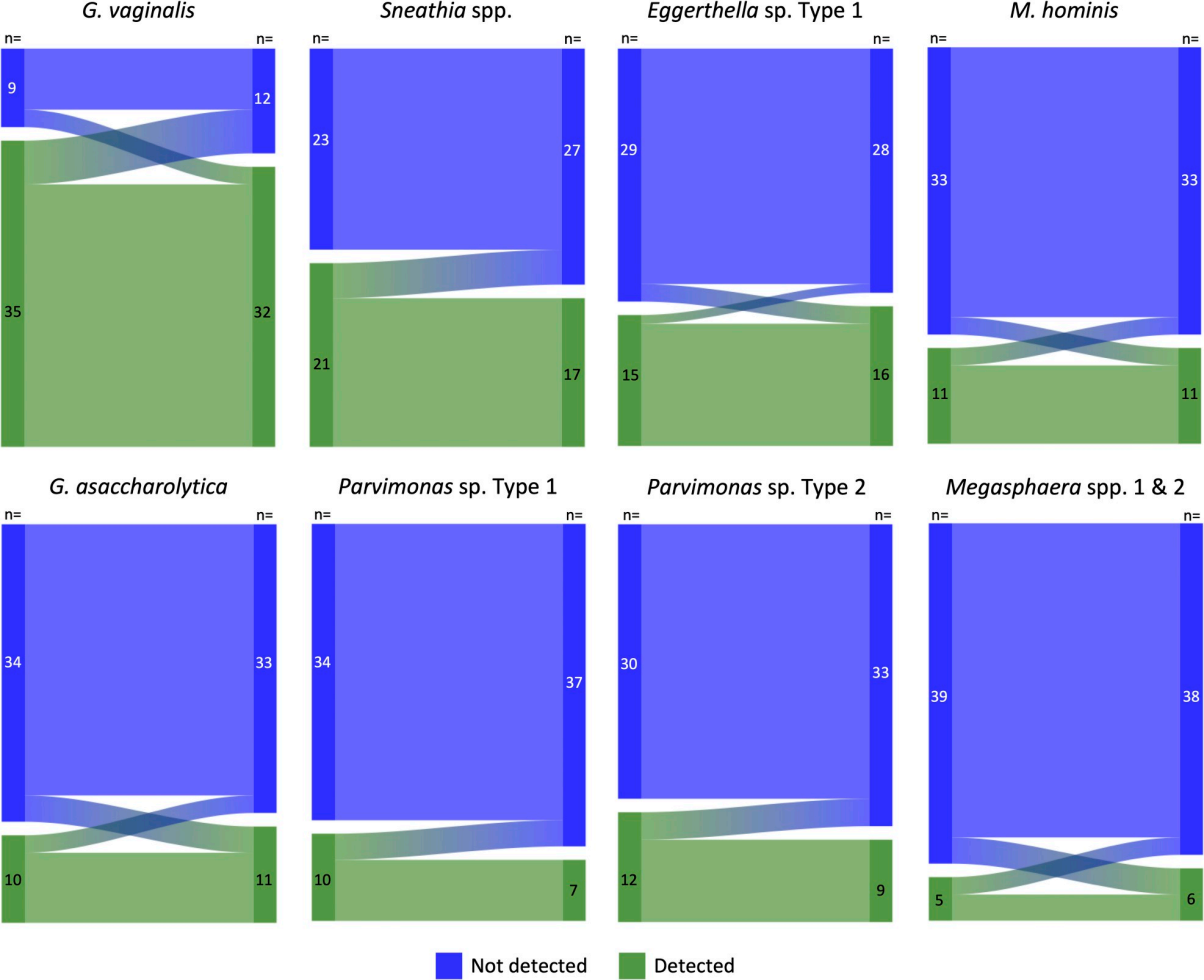
Change in presence/absence of the eight bacterial taxa from enrollment to the three-month follow-up visit among the 44 women who returned for the follow-up visit. Enrollment: visit DMPA-IM was administered; Follow-up: visit three-months post DMPA-IM.

All eight taxa assessed were found in this group of postpartum women, however only *G*. *vaginalis* was detectable in a majority of participants at enrollment (non-HC: 71%; DMPA-IM: 85%) and follow-up (non-HC: 85%; DMPA-IM: 73%); the other seven taxa were detectable in fewer than 50% of women (Table 3). There was no difference in the detectability of the eight taxa between the two contraceptive groups at enrollment, nor was there any significant difference in mean concentration of each taxon among women with the taxon detected, with the exception of *G*. *asaccharolytica* which was found at a slightly higher concentration among non-HC users at enrollment (p = 0.037). Women had an average of 3.0 taxa (SD 2.5) present at enrollment and 2.5 (SD 2.4) at the three-month follow-up visit, neither of which were significantly different between contraceptive groups (p = 0.609 and p = 0.653, respectively) or from baseline to follow-up for either contraceptive group (non-HC: p = 0.253; DMPA-IM: p = 0.678) (S1 Fig). Within contraceptive group, detection of each taxon at follow-up was not significantly different from detection at enrollment (S1 Table). Qualitatively, very few women went from undetectable to detectable, or vice-versa, from enrollment to follow-up for each of the eight selected bacterial taxa (Fig 1).

**Table 3:** Populations of key vaginal bacteria in postpartum women at enrollment and three-months post-initiation of DMPA-IM or non-hormonal contraception.

	**Detectability at Enrollment^a^**			**Concentration among Women with Taxon Detected at Enrollment^b^^,^^c^**		
**Taxa**	**Non-HC (n = 21)**	**DMPA-IM (n = 33)**	**P-value^d^**	**Non-HC**	**DMPA-IM**	**P-value^e^**
Total bacterial load	--	--		8.31 (0.84)	8.59 (0.58)	0.158
*G*. *vaginalis*	15 (71.4)	28 (84.9)	0.233	6.86 (1.61)	7.43 (1.69)	0.294
*Sneathia* spp.	11 (52.4)	15 (45.5)	0.619	6.68 (1.88)	5.69 (1.89)	0.199
*Eggerthella* sp. Type 1	9 (42.9)	12 (36.4)	0.633	6.58 (1.78)	7.17 (1.54)	0.431
*M*. *hominis*	9 (42.9)	7 (21.2)	0.089	6.66 (1.81)	5.10 (1.72)	0.103
*G*. *asaccharolytica*	6 (28.6)	11 (33.3)	0.713	6.95 (1.09)	5.72 (1.04)	0.037
*Parvimonas* sp. Type 1	8 (38.1)	6 (18.2)	0.104	5.77 (2.27)	4.61 (1.58)	0.308
*Parvimonas* sp. Type 2	7 (33.3)	11 (33.3)	1.000	6.72 (2.19)	5.93 (2.09)	0.454
*Megasphaera* spp. 1 & 2	3 (14.3)	5 (15.2)	1.000	6.09 (3.08)	7.53 (0.38)	0.315
	**Detectability at Follow-up**^**a**^			**Concentration among Women with Taxon Detected at Follow-up**^**b**^^,^^**c**^		
**Taxa**^**a,b**^	**Non-HC (n = 18)**	**DMPA-IM (n = 26)**	**P-value**^**d**^	**Non-HC**	**DMPA-IM**	**P-value**^**e**^
Total bacterial load	--	--		8.74 (0.50)	8.31 (0.89)	0.072
*G*. *vaginalis*	13 (72.2)	19 (73.1)	0.950	7.06 (1.52)	7.24 (1.42)	0.735
*Sneathia* spp.	6 (33.3)	11 (42.3)	0.548	6.14 (2.10)	5.11 (2.31)	0.379
*Eggerthella* sp. Type 1	6 (33.3)	10 (38.5)	0.728	6.71 (2.08)	6.03 (2.29)	0.562
*M*. *hominis*	5 (27.8)	6 (23.1)	0.723	5.94 (2.64)	5.28 (1.55)	0.620
*G*. *asaccharolytica*	3 (16.7)	8 (30.8)	0.480	6.30 (2.32)	5.05 (1.76)	0.357
*Parvimonas* sp. Type 1	2 (11.1)	5 (19.2)	0.682	6.77 (1.99)	3.70 (0.83)	0.025
*Parvimonas* sp. Type 2	4 (22.2)	5 (19.2)	1.000	5.70 (3.19)	6.11 (2.41)	0.831
*Megasphaera* spp. 1 & 2	2 (11.1)	4 (15.4)	1.000	4.21 (2.89)	5.60 (2.43)	0.564

Abbreviations: DMPA-IM, intramuscular depot-medroxyprogesterone acetate; HC, hormonal contraception; SD, standard deviation.

Enrollment: visit DMPA-IM was administered; Follow-up: visit three-months post DMPA-IM.

^a^ Data displayed as n (%).

^b^ Concentration displayed as log_10_ copies/swab.

^c^ Data displayed as mean (SD).

^d^ Chi-square test or Fisher's exact test (for comparisons containing cells with <5 observations).

^e^ T-test.

### Patterns of vaginal microbiota change among intramuscular depot-medroxyprogesterone acetate users compared to non-hormonal contraceptive users

**Fig 2:**
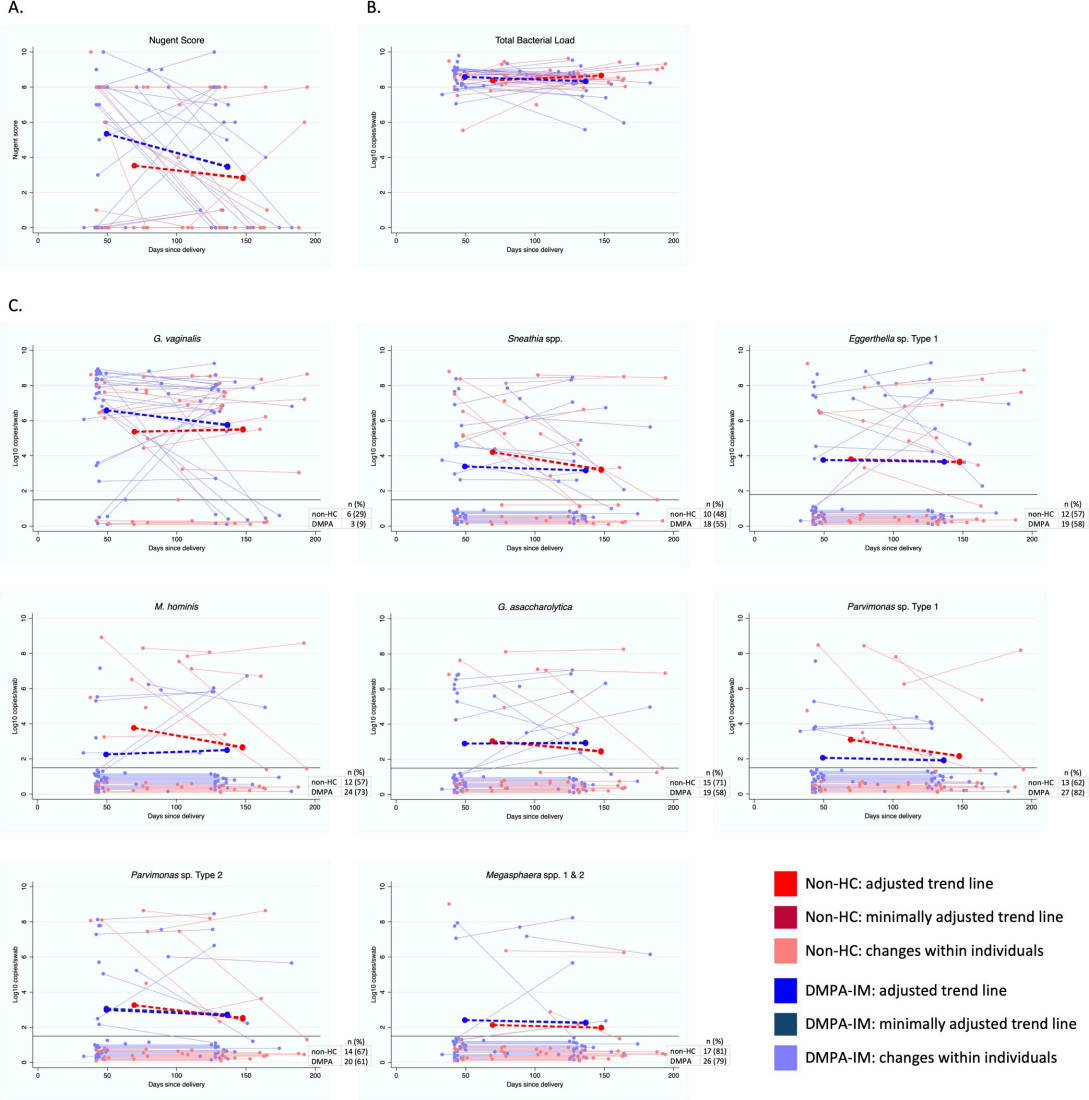
**(A) Nugent score, (B) total bacterial load, and (C) concentration of vaginal taxa over time, by contraceptive group, with fitted trend lines for mean change among all women.** Abbreviations: DMPA-IM, intramuscular depot-medroxyprogesterone acetate; HC, hormonal contraception. Bacterial concentrations were log_10_ transformed to normalize their distribution. All values below LLD (black horizontal bar on graph) were equal, but values were jittered to allow for visualization of all observations. Trend lines for mean change within each contraceptive group were estimated using GEE with an interaction term between contraceptive group and days from delivery to enrollment and adjusted for days from enrollment to vaginal swab collection; trend lines show mean enrollment and exit dates for each group.

Over three months, mean Nugent score decreased by 1.90 points (95%CI: -3.38 to -0.41, p = 0.012) among women using DMPA-IM and by 0.73 points (95%CI: -2.70 to 1.24, p = 0.469) among women using non-HC, resulting in similar mean Nugent scores in both contraceptive groups at follow-up (Table 4, Fig 2A). The difference in change between the two contraceptive groups was not statistically significant, however (p = 0.354). In a small subset of women, the opposite pattern was observed: these women maintained an elevated Nugent score or increased in Nugent score after enrollment. Maintenance of an elevated Nugent score or an increase in Nugent score after enrollment was observed mostly among women using DMPA-IM (n = 5 [19%] DMPA-IM users vs. n = 2 [11%] non-HC users). Non-HC users started with lower Nugent scores, and were less likely to change Nugent category (S2 Fig). Total bacterial load decreased non-significantly among DMPA-IM users and increased non-significantly among non-HC users over follow-up, resulting in a significant difference in changes of total bacterial load between the two contraceptive groups (difference in Δ = -0.58 log_10_ copies/swab [95%CI: 1.16 to -0.01], p = 0.049) (Table 4, Fig 2B).

**Table 4:** Change in Nugent score, total bacterial load, and concentration of vaginal taxa over time by contraceptive group, and difference in change between contraceptive groups during follow-up.

**All participants^a^**										
		**non-HC**	**95% CI**	**P-value**	**DMPA-IM**	**95% CI**	**P-value**	**Difference**	**95% CI**	**P-value**
Nugent score		-0.73	-2.70, 1.24	0.469	-1.90	-3.38, -0.41	**0.012**	-1.17	-3.64, 1.30	0.354
Total 16S rRNA		0.33	-0.13, 0.79	0.160	-0.25	-0.60, 0.10	0.159	-0.58	-1.16, -0.01	**0.049**
*G*. *vaginalis*		0.22	-1.02, 1.46	0.725	-0.87	-1.81, 0.06	0.067	-1.10	-2.65, 0.45	0.166
*Sneathia* spp.		-1.09	-2.05, -0.13	**0.026**	-0.23	-0.96, 0.49	0.533	0.86	-0.34, 2.06	0.161
*Eggerthella* sp. Type 1		-0.12	-1.18, 0.94	0.827	-0.06	-0.87, 0.74	0.875	0.05	-1.28, 1.38	0.936
*M*. *hominis*		-1.21	-2.11, -0.3	**0.009**	0.29	-0.39, 0.98	0.400	1.50	0.37, 2.63	**0.010**
*G*. *asaccharolytica*		-0.63	-1.53, 0.28	0.174	0.06	-0.62, 0.75	0.855	0.69	-0.44, 1.82	0.233
*Parvimonas* sp. Type 1		-1.03	-1.82, -0.24	**0.010**	-0.11	-0.70, 0.49	0.722	0.92	-0.07, 1.91	0.067
*Parvimonas* sp. Type 2		-0.80	-1.79, 0.18	0.110	-0.26	-1.03, 0.50	0.501	0.54	-0.71, 1.79	0.397
*Megasphaera* spp. 1 & 2		-0.13	-0.99, 0.72	0.758	-0.15	-0.80, 0.49	0.640	-0.02	-1.09, 1.05	0.972
Participants with ≥1 detectable value during follow-up^b^										
	n	non-HC	95% CI	P-value	DMPA-IM	95% CI	P-value	Difference	95% CI	P-value
*G*. *vaginalis*	47	0.39	-1.30, 2.09	0.647	-1.01	-2.12, 0.10	0.075	-1.40	-3.42, 0.62	0.174
*Sneathia* spp.	26	-2.19	-3.74, -0.63	**0.006**	-0.62	-1.85, 0.62	0.327	1.57	-0.41, 3.56	0.121
*Eggerthella* sp. Type 1	23	-0.27	-2.36, 1.82	0.801	-0.30	-1.94, 1.33	0.716	-0.03	-2.69, 2.62	0.980
*M*. *hominis*	18	-3.06	-4.99, -1.13	**0.002**	1.24	-0.63, 3.11	0.194	4.30	1.61, 6.99	**0.002**
*G*. *asaccharolytica*	20	-1.96	-4.18, 0.26	0.084	0.14	-1.30, 1.58	0.848	2.10	-0.55, 4.74	0.120
*Parvimonas* sp. Type 1	14	-2.50	-4.63, -0.36	**0.022**	-0.92	-3.21, 1.38	0.435	1.58	-1.56, 4.72	0.323
*Parvimonas* sp. Type 2	20	-2.71	-5.35, -0.07	**0.045**	-0.56	-2.41, 1.29	0.552	2.14	-1.08, 5.37	0.193
*Megasphaera* spp. 1 & 2	11	-0.91	-4.75, 2.94	0.644	-0.80	-3.31, 1.71	0.535	0.11	-4.48, 4.71	0.962

Abbreviations: CI, confidence interval; DMPA-IM, intramuscular depot-medroxyprogesterone acetate; HC, hormonal contraception.

Enrollment: visit DMPA-IM was administered; Follow-up: visit three-months post DMPA-IM.

Concentration displayed as log_10_ copies/swab. All values below LLD were set to half the LLD for that assay.

^a^ Mean change for each contraceptive group and difference in change estimated using GEE with an interaction term between contraceptive group and days from enrollment to vaginal swab collection and adjusted for days from delivery to enrollment, age, marital status, and resumption of intercourse at enrollment.

^b^ Mean change for each contraceptive group and difference in change among women with ≥1 detectable value for that taxon during follow-up estimated using GEE with an interaction term between contraceptive group and days from enrollment to vaginal swab collection and adjusted for days from delivery to enrollment.

In analyses including all women, the mean concentration of *G*. *vaginalis* decreased by 0.87 log_10_ copies/swab (95%CI: -1.81 to 0.06, p = 0.067) among DMPA-IM users and increased by 0.22 (95%CI: -1.02 to 1.46, p = 0.725) among non-HC users (Table 4, Fig 2C). As was seen with Nugent score, there was a small subset of DMPA-IM users (n = 5 [19%]) who did not follow the overall trend; this subset was characterized by an increase in *G*. *vaginalis* concentration of ≥1 log_10_ copies/swab. Of these 5 women with a pattern of increasing *G*. *vaginalis*, 3 (60%) also exhibited the pattern of increasing Nugent score.

The concentration of three of the bacterial taxa assessed decreased significantly among women using non-HC over follow-up. *Sneathia* spp. (Δ = -1.09 log_10_ copies/swab [95%CI: -2.05 to -0.13], p = 0.026), *M*. *hominis* (Δ = -1.21 log_10_ copies/swab [95%CI: -2.11 to -0.30], p = 0.009), and *Parvimonas* sp. Type 1 (Δ = -1.03 log_10_ copies/swab [95%CI: -1.82 to -0.24], p = 0.010) all decreased by ≥1 log_10_ copies/swab on average (Table 4, Fig 2C). In contrast, concentrations were more stable among DMPA-IM users, with non-significant changes (*Sneathia* spp.: Δ = -0.23 log_10_ copies/swab [95%CI: -0.96 to 0.49], p = 0.533; *M*. *hominis*: Δ = 0.29 log_10_ copies/swab [95%CI: -0.39 to 0.98], p = 0.400; and *Parvimonas* sp. Type 1: Δ = -0.11 log_10_ copies/swab [95%CI: -0.70 to 0.49], p = 0.722). The observed changes in concentration over follow-up were only statistically significantly different between contraceptive groups for *M*. *hominis* (difference in Δ = 1.50 [95%CI: 0.37 to 2.63], p = 0.010). In sensitivity analyses limited to women with at least one detectable bacterial taxon during follow-up, we observed the same patterns as above, however the changes were more pronounced (decrease of ≥2 log_10_ copies/swab on average) compared to the analysis that included all participants (Table 4, S3 Fig).

### Patterns of vaginal microbiota change among intramuscular depot-medroxyprogesterone acetate users only

**Fig 3:**
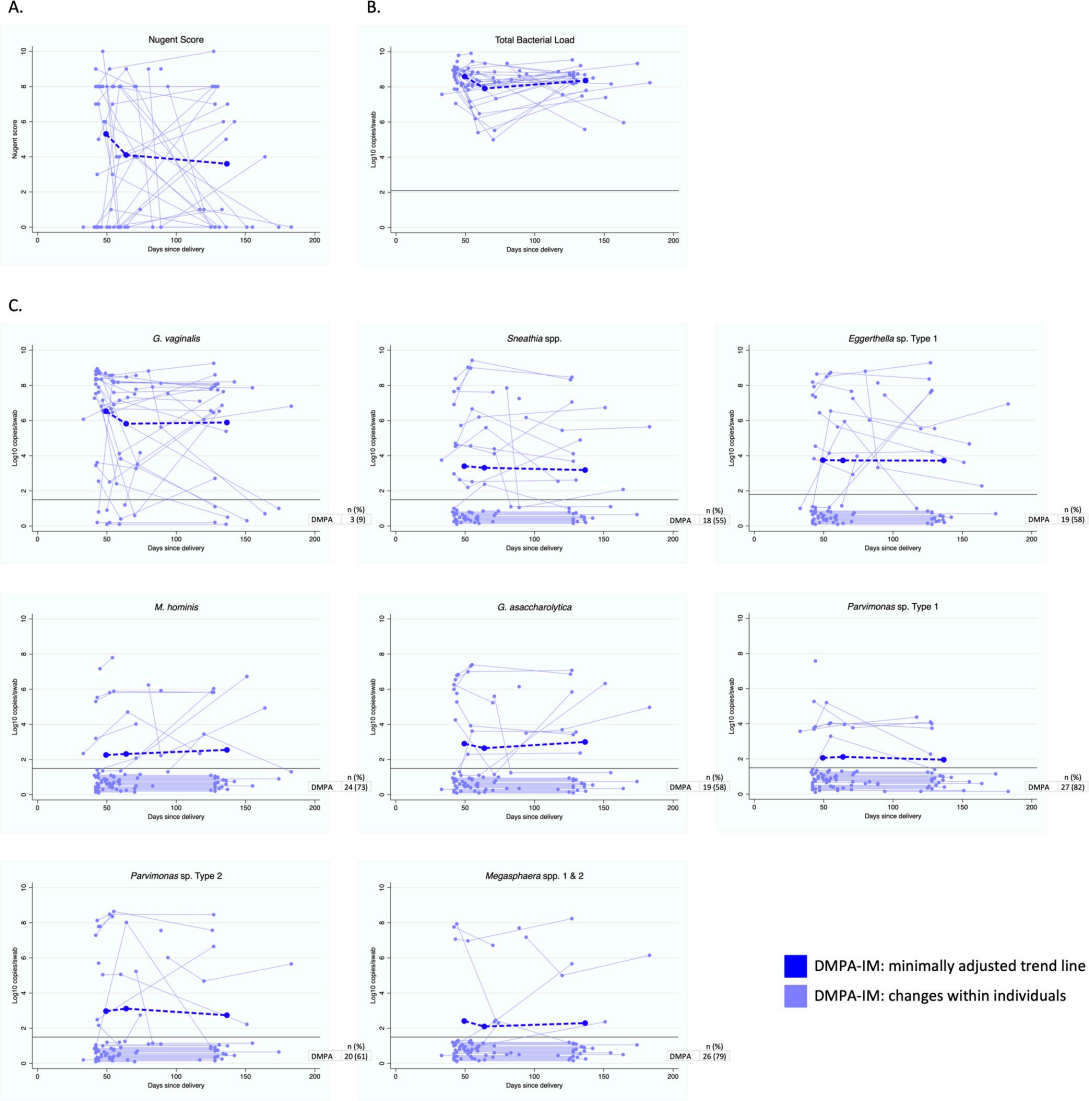
**(A) Nugent score, (B) total bacterial load, and (C) concentration of vaginal taxa over time among DMPA-IM users only with fitted trend lines for mean change among all women.** Abbreviations: DMPA-IM, intramuscular depot-medroxyprogesterone acetate. Bacterial concentrations were log_10_ transformed to normalize their distribution. All values below LLD (black horizontal bar on graph) were equal, but values were jittered to allow for visualization of all observations. Trend lines for mean change were estimated using GEE adjusted for days from delivery to enrollment and days from enrollment to vaginal swab collection; trend lines show mean enrollment and exit dates.

In the DMPA-IM group, specimens were collected during the time when serum MPA levels are known to peak (9–14 days post-injection) [34, 35]. The median number of days between enrollment and the two-week follow-up visit was 12 days (IQR: 9–22 days). There were no significant patterns in change for Nugent score or the eight bacterial taxa in the two time periods post DMPA-IM injection (Table 5, Fig 3, S4 Fig). Total bacterial load decreased significantly in the first two weeks post-injection (enrollment to two-week visit: Δ = -0.64 [95%CI: -1.03 to -0.25]; p = 0.001) but rebounded by the 3-month follow-up visit (two-week visit to three-month visit: Δ = 0.44 [95%CI: 0.02 to 0.86]; p = 0.041).

**Table 5:** Change in Nugent score, total bacterial load, and concentration of vaginal taxa over time among DMPA-IM users only, and difference in change between time periods.

**All participants^a^**										
****	****	**Enrollment to 2-week visit**	**95% CI**	**P-value**	**2-week visit to 3-month visit**	**95% CI**	**P-value**	**Difference**	**95% CI**	**P-value**
Nugent score		-1.31	-2.74, 0.12	0.073	-0.62	-2.17, 0.93	0.433	0.69	-1.90, 3.27	0.601
Total 16S rRNA		-0.64	-1.03, -0.25	**0.001**	0.44	0.02, 0.86	**0.041**	1.08	0.37, 1.78	**0.003**
*G*. *vaginalis*		-0.66	-1.55, 0.23	0.146	-0.22	-1.18, 0.74	0.658	0.44	-1.16, 2.04	0.589
*Sneathia* spp.		-0.09	-0.67, 0.49	0.761	-0.06	-0.70, 0.57	0.846	0.03	-1.03, 1.08	0.959
*Eggerthella* sp. Type 1		0.01	-0.75, 0.77	0.974	-0.06	-0.88, 0.76	0.882	-0.07	-1.44, 1.29	0.914
*M*. *hominis*		0.03	-0.49, 0.55	0.915	0.33	-0.23, 0.90	0.248	0.30	-0.63, 1.24	0.526
*G*. *asaccharolytica*		-0.19	-0.76, 0.37	0.500	0.37	-0.24, 0.98	0.236	0.57	-0.46, 1.59	0.278
*Parvimonas* sp. Type 1		0.06	-0.28, 0.39	0.741	-0.19	-0.55, 0.18	0.312	-0.24	-0.85, 0.36	0.431
*Parvimonas* sp. Type 2		0.15	-0.49, 0.79	0.647	-0.40	-1.12, 0.32	0.275	-0.55	-1.74, 0.64	0.364
*Megasphaera* spp. 1 & 2		-0.23	-0.73, 0.26	0.357	0.16	-0.37, 0.70	0.550	0.40	-0.50, 1.29	0.384
**Participants with ≥1 detectable value during follow-up**^**b**^										
	**n**	**Enrollment to 2-week visit**	**95% CI**	**P-value**	**2-week visit to 3-month visit**	**95% CI**	**P-value**	**Difference**	**95% CI**	**P-value**
*G*. *vaginalis*	31	-0.68	-1.65, 0.29	0.167	-0.24	-1.28, 0.81	0.658	0.45	-1.30, 2.19	0.617
*Sneathia* spp.	16	-0.23	-1.22, 0.76	0.651	0.13	-1.05, 1.32	0.825	0.36	-1.48, 2.21	0.700
*Eggerthella* sp. Type 1	16	-0.08	-1.54, 1.37	0.910	-0.27	-2.04, 1.51	0.769	-0.18	-2.93, 2.57	0.897
*M*. *hominis*	10	0.05	-1.34, 1.44	0.943	1.48	-0.09, 3.04	0.064	1.43	-1.15, 4.00	0.278
*G*. *asaccharolytica*	14	-0.31	-1.12, 0.50	0.456	1.39	0.46, 2.32	**0.003**	1.70	0.22, 3.19	**0.025**
*Parvimonas* sp. Type 1^c^	7	--			--			--		
*Parvimonas* sp. Type 2	17	0.54	-0.71, 1.79	0.397	-1.19	-2.70, 0.32	0.124	-1.73	-4.13, 0.68	0.159
*Megasphaera* spp. 1 & 2^c^	8	--			--			--		

Abbreviations: CI, confidence interval; DMPA-IM, intramuscular depot-medroxyprogesterone acetate.

Enrollment: visit DMPA-IM was administered; two-week visit: visit 14 days post DMPA-IM; Follow-up: visit three-months post DMPA-IM.

Concentration displayed as log_10_ copies/swab. All values below LLD were set to half the LLD for that assay.

^a^ Mean change in the two time periods and difference in change between time periods estimated using GEE adjusted for days from enrollment to vaginal swab collection and days from delivery to enrollment.

^b^ Mean change in the two time periods and difference in change between time periods among women with ≥1 detectable value for that taxon during follow-up estimated using GEE adjusted for days from enrollment to vaginal swab collection and days from delivery to enrollment.

^c^ Too few observations to model data.

## Discussion

Our study shows that vaginal bacteria previously associated with HIV acquisition were present in African women initiating postpartum contraception, although no single taxon was found in a majority of women. Findings suggested differential patterns of change in three of the eight taxa assessed, *Sneathia* spp., *M*. *hominis*, and *Parvimonas* sp. Type 1, among women initiating and using DMPA-IM compared to non-HC; the concentration of these three taxa decreased among women using non-HC but no change in concentration was observed among women using DMPA-IM. While significant decreases in concentrations of *Sneathia* spp., *M*. *hominis*, and *Parvimonas* sp. Type 1 were observed among non-HC users, there were only significantly different changes in the concentration of *M*. *hominis* between users of DMPA-IM and non-HC. Observed patterns in change were especially pronounced among women with these bacteria present at contraception initiation. DMPA-IM use did not increase the likelihood of detection or the concentration of these bacteria, and changes in bacterial concentrations were not correlated with the timing of peak plasma MPA levels.

There are several potential explanations for the observed stability in *Sneathia* spp., *M*. *hominis*, and *Parvimonas* sp. Type 1 concentrations at the three-month follow-up visit observed among DMPA-IM users. Recent studies of the vaginal microbiome in pregnancy and the postpartum period have found that the vaginal microbiota in the one to six weeks after delivery are much more diverse, with lower levels of *Lactobacillus* spp., compared to both pre-pregnancy and during pregnancy [43, 44]. This time also coincides with a known period of higher HIV risk in women [45]. DMPA-IM use could stabilize concentrations of vaginal anerobic bacteria in the postpartum period by allowing a hypoestrogenic state to persist, while non-HC users returned to the more optimal vaginal bacterial communities seen in pre-pregnancy as their normal hormonal cycles return. Low levels of estrogen have been associated with glycogen suppression [8, 14], and glycogen is an important substrate for *Lactobacillus* species [14–16]. Consequently, a reduction of glycogen in the female reproductive tract could maintain anaerobic, non-optimal vaginal microbiota. This explanation aligns well with our observations: DMPA-IM initiation was not associated with large increases in concentration of the seven bacterial taxa linked to HIV acquisition in our study, however it appears that DMPA-IM might support continued higher concentrations compared to non-HC. Alternatively, however, this pattern could be due to the fact that participants selected their own contraceptive method, and women who rely on non-hormonal methods alone appear to be behaviorally and demographically different from women who choose highly effective contraceptive methods [46], including in sexual and personal hygiene practices. We collected information on potential confounders to enable for control of this confounding in statistical analyses, however some measures relied on self-report and are likely imperfect.

Our results generally agree with recent findings from a study in Zimbabwe conducted among HIV-negative, nonpregnant women [27]. Achilles et al. reported that use of DMPA-IM did not significantly change the concentration of three common BV-associated species: *G*. *vaginalis*, *Atopobium vaginae*, and *Megasphaera*-like bacterium phylotype 1. Unlike the Zimbabwean cohort, we did observe a significant decrease in Nugent score among the DMPA-IM users in our study, which is congruent with existing literature [47]. Women who chose DMPA-IM in our study had much higher Nugent scores at enrollment compared to women who chose non-HC, however, giving them more room to decrease in Nugent score and posing a concern that these groups of women were not completely comparable. This discrepancy in Nugent score at enrollment may be explained, at least in part, by the fact that more DMPA-IM users had resumed sexual intercourse prior to enrollment.

Strengths of this study include that we enrolled participants who were free of any hormonal contraceptives prior to enrollment and received their first DMPA-IM injection from study staff. This design eliminated contamination from other contraceptives, as well as uncertainty in type of injectable hormonal contraceptive used due to self-report. It also allowed for comparison of pre- and post-DMPA-IM effects, as women could serve as their own controls. Another strength is that specimens were collected during the time period when MPA levels peak [34, 35], allowing for the examination of a relationship between vaginal microbiota and high MPA plasma levels. Furthermore, our study focused on African women, the geographical population at highest risk of STI/HIV acquisition from DMPA-IM use. Lastly, we used qPCR to measure the absolute abundance of vaginal bacteria, rather than relying on relative abundance measures.

Our study was limited in power because most women had concentrations below the lower limit of detection for each taxon, and most women remained in the same detection category in which they started; this restricted our analytic options and some models contained relatively few women. This limitation exemplifies the complexities of working with qPCR data. Additionally, we had a modest loss to follow up (19% of women), further reducing our power to detect true differences. A second limitation is that participants selected their own contraceptive method, and women choosing non-HC may not represent a counterfactual population for women who choose DMPA-IM. Also, women who chose non-HC did not return for a visit 9–14 days after enrollment since their vaginal microbiota profiles were not expected to change in the two weeks following enrollment, resulting in lack of comparable data at this time point. Our study may also be limited in generalizability due to the source population of our participants. Postpartum, amenorrheic women were enrolled to reduce the variability of the vaginal microbiome due to the menstrual cycle [9, 17, 33], however vaginal microbiota in the postpartum period may differ from vaginal microbiota found in menstruating women of childbearing age [43, 44]. Lastly, the use of a qPCR, a targeted approach, might have missed changes in important bacterial taxa not assessed, including taxa associated with an optimal vaginal microbiota. This is an area that future work might address.

Interestingly, there was a small but notable subset of DMPA-IM users that did not follow overall trends and maintained or increased in Nugent score and concentration of *G*. *vaginalis*. We looked within this subgroup for commonalities, and while we could not find any demographic, behavioral, or physical similarities, including changes in vaginal bleeding patterns and perturbation of the regular menstrual cycle, among these women from the data we collected, there is potentially a subgroup of women for which DMPA-IM has a different effect. While small, this subgroup could contribute to the increased risk of HIV seen in observational studies among DMPA-IM users. Research now suggests that there are likely at least four sub-strains/genotypes of *G*. *vaginalis*, not all of which produce sialidase, and which may have independent virulence factors contributing to symptoms and/or sequelae of vaginosis [48–50]. One possible area for future investigation is the relationship between DMPA-IM and sialidase-producing sub-strains of *G*. *vaginalis* to see if DMPA-IM has a differential effect on more pathogenic *G*. *vaginalis*.

Overall, these findings suggest that bacteria associated with increased HIV acquisition risk are present among postpartum women at average risk for HIV acquisition in Kenya, and that DMPA-IM use, compared to non-HC user, showed differential patterns in concentration change with three taxa associated with HIV acquisition. Our findings indicate that DMPA-IM use may support an anaerobic vaginal microbiota associated with HIV acquisition, especially among a subset of women with these species already present. High alpha- and beta-diversity may also remain for a longer period in postpartum DMPA-IM initiators, a question we plan to explore in broad range microbiome data from this cohort. While our numbers were small and we did not have as much power as we planned, these findings suggest postpartum use of DMPA-IM deserves further attention; DMPA-IM use in the postpartum period may pose different risks than DMPA-IM use in non-postpartum women of reproductive age, and more research on the effect of contraception initiation on the vaginal microbiome in this subgroup of women is warranted. While the ECHO trial has relieved apprehension about use of DMPA-IM in women in high HIV prevalence settings, specific subpopulations may still be at increased risk of HIV; DMPA-IM use in the postpartum period may pose a different risk than it does in the general population, due to high vaginal microbial diversity seen in this period. Planned microbiome studies from our cohort and among women in the ECHO trial may help to further answer questions about how DMPA-IM influences vaginal bacteria.

## Supporting information

S1 File Supplemental Methods: DNA extraction, DNA quantification, and polymerase chain reaction. (DOCX)

S1 Table McNemar’s test for change in Nugent-BV category and detectability of bacterial taxa from enrollment to three-month follow-up by contraceptive group. Abbreviations: BV, bacterial vaginosis; DMPA-IM, intramuscular depot-medroxyprogesterone acetate; HC, hormonal contraception.
Enrollment: visit DMPA-IM was administered; Follow-up: visit three-months post DMPA-IM.
^a^ Exact McNemar significance probability.
^b^ No switches in detectability.
(DOCX)

S1 Fig **Histogram of the number of taxa present (out of the eight assessed) at (A) Enrollment (n = 54) and (B) the three-month follow-up visit (n = 44).**
Abbreviations: DMPA-IM, intramuscular depot-medroxyprogesterone acetate; HC, hormonal contraception.
Enrollment: visit DMPA-IM was administered; Follow-up: visit three-months post DMPA-IM.
(TIF)

S2 Fig **Change in Nugent category from enrollment to the three-month follow-up for (A) non-HC users (n = 18) and (B) DMPA-IM users (n = 26), among the 44 women who returned for follow-up. (C) Change in Nugent score category from enrollment to the two-week post-injection visit to the three-month follow-up visit among DMPA-IM users (n = 26) who attended every visit.**
Abbreviations: DMPA-IM, intramuscular depot-medroxyprogesterone acetate; HC, hormonal contraception.
Enrollment: visit DMPA-IM was administered; two-week visit: visit 14 days post DMPA-IM; Follow-up: visit three-months post DMPA-IM.
(TIF)

S3 Fig **(A) Nugent score, (B) total bacterial load, and (C) concentration of vaginal taxa over time, by contraceptive group, with fitted trend lines for mean change among women ≥1 detectable value during follow-up.**
Abbreviations: DMPA-IM, intramuscular depot-medroxyprogesterone acetate; HC, hormonal contraception.
Bacterial concentrations were log_10_ transformed to normalize their distribution.
All values below LLD (black horizontal bar on graph) were equal, but values were jittered to allow for visualization of all observations.
Trend lines for mean change in concentration among women ≥1 detectable value during follow-up within each contraceptive group were estimated using GEE with an interaction term between contraceptive group and days from delivery to enrollment and adjusted for days from enrollment to vaginal swab collection; trend lines show mean enrollment and exit dates for each group.
(TIF)

S4 Fig **(A) Nugent score, (B) total bacterial load, and (C) concentration of vaginal taxa over time among DMPA-IM users only with fitted trend lines for mean change among women ≥1 detectable value during follow-up.**
Abbreviations: DMPA-IM, intramuscular depot-medroxyprogesterone acetate.
Bacterial concentrations were log_10_ transformed to normalize their distribution.
All values below LLD (black horizontal bar on graph) were equal, but values were jittered to allow for visualization of all observations.
Trend lines for mean change in concentration among women ≥1 detectable value during follow-up were estimated using GEE adjusted for days from delivery to enrollment and days from enrollment to vaginal swab collection; trend lines show mean enrollment and exit dates.
(TIF)

## References

[1] Ravel J, Gajer P, Abdo Z, Schneider GM, Koenig SS, McCulle SL, et al Vaginal microbiome of reproductive-age women. Proc Natl Acad Sci U S A. 2011;108 Suppl 1:4680–7. Epub 2010/06/11. 10.1073/pnas.1002611107 20534435 PMC3063603

[2] Smith SB, Ravel J. The vaginal microbiota, host defence and reproductive physiology. J Physiol. 2017;595(2):451–63. Epub 2016/07/05. 10.1113/JP271694 27373840 PMC5233653

[3] Anahtar MN, Byrne EH, Doherty KE, Bowman BA, Yamamoto HS, Soumillon M, et al Cervicovaginal bacteria are a major modulator of host inflammatory responses in the female genital tract. Immunity. 2015;42(5):965–76. Epub 2015/05/21. 10.1016/j.immuni.2015.04.019 25992865 PMC4461369

[4] Brotman RM. Vaginal microbiome and sexually transmitted infections: an epidemiologic perspective. J Clin Invest. 2011;121(12):4610–7. Epub 2011/12/03. 10.1172/JCI57172 22133886 PMC3225992

[5] Gosmann C, Anahtar MN, Handley SA, Farcasanu M, Abu-Ali G, Bowman BA, et al Lactobacillus-Deficient Cervicovaginal Bacterial Communities Are Associated with Increased HIV Acquisition in Young South African Women. Immunity. 2017;46(1):29–37. Epub 2017/01/15. 10.1016/j.immuni.2016.12.013 28087240 PMC5270628

[6] McClelland RS, Lingappa JR, Srinivasan S, Kinuthia J, John-Stewart GC, Jaoko W, et al Evaluation of the association between the concentrations of key vaginal bacteria and the increased risk of HIV acquisition in African women from five cohorts: a nested case-control study. Lancet Infect Dis. 2018;18(5):554–64. Epub 2018/02/06. 10.1016/S1473-3099(18)30058-6 29396006 PMC6445552

[7] Brotman RM, Ravel J, Bavoil PM, Gravitt PE, Ghanem KG. Microbiome, sex hormones, and immune responses in the reproductive tract: challenges for vaccine development against sexually transmitted infections. Vaccine. 2014;32(14):1543–52. Epub 2013/10/19. 10.1016/j.vaccine.2013.10.010 24135572 PMC3964794

[8] Hapgood JP, Kaushic C, Hel Z. Hormonal Contraception and HIV-1 Acquisition: Biological Mechanisms. Endocr Rev. 2018;39(1):36–78. Epub 2018/01/09. 10.1210/er.2017-00103 29309550 PMC5807094

[9] Srinivasan S, Liu C, Mitchell CM, Fiedler TL, Thomas KK, Agnew KJ, et al Temporal variability of human vaginal bacteria and relationship with bacterial vaginosis. PLoS One. 2010;5(4):e10197 Epub 2010/04/27. 10.1371/journal.pone.0010197 20419168 PMC2855365

[10] Trends in Contraceptive Use Worldwide 2015. United Nations, 2015.

[11] van de Wijgert J, Jespers V. The global health impact of vaginal dysbiosis. Res Microbiol. 2017;168(9–10):859–64. Epub 2017/03/05. 10.1016/j.resmic.2017.02.003 . 28257809

[12] UNAIDS data 2018. UNAIDS: UNAIDS, 2018.

[13] American College of Obstetricians and Gynecologists CoAHC, Committee on Gynecologic Practice. Depot medroxyprogesterone acetate and bone effects. Committee Opinion No. 602. Obstet Gynecol. 2014;(123):1398–402. 24848921 10.1097/01.AOG.0000450758.95422.c8

[14] Miller L, Patton DL, Meier A, Thwin SS, Hooton TM, Eschenbach DA. Depomedroxyprogesterone-induced hypoestrogenism and changes in vaginal flora and epithelium. Obstet Gynecol. 2000;96(3):431–9. Epub 2000/08/29. 10.1016/s0029-7844(00)00906-6 . 10960638

[15] Mirmonsef P, Hotton AL, Gilbert D, Burgad D, Landay A, Weber KM, et al Free glycogen in vaginal fluids is associated with Lactobacillus colonization and low vaginal pH. PLoS One. 2014;9(7):e102467 Epub 2014/07/18. 10.1371/journal.pone.0102467 25033265 PMC4102502

[16] Mirmonsef P, Modur S, Burgad D, Gilbert D, Golub ET, French AL, et al Exploratory comparison of vaginal glycogen and Lactobacillus levels in premenopausal and postmenopausal women. Menopause. 2015;22(7):702–9. Epub 2014/12/24. 10.1097/GME.0000000000000397 25535963 PMC4476965

[17] Gajer P, Brotman RM, Bai G, Sakamoto J, Schutte UM, Zhong X, et al Temporal dynamics of the human vaginal microbiota. Sci Transl Med. 2012;4(132):132ra52. Epub 2012/05/04. 10.1126/scitranslmed.3003605 22553250 PMC3722878

[18] Brind J, Condly SJ, Mosher SW, Morse AR, Kimball J. Risk of HIV Infection in Depot-Medroxyprogesterone Acetate (DMPA) Users: A Systematic Review and Meta-analysis. Issues Law Med. 2015;30(2):129–39. Epub 2015/12/30. . 26710371

[19] Morrison CS, Chen PL, Kwok C, Baeten JM, Brown J, Crook AM, et al Hormonal contraception and the risk of HIV acquisition: an individual participant data meta-analysis. PLoS Med. 2015;12(1):e1001778 Epub 2015/01/23. 10.1371/journal.pmed.1001778 25612136 PMC4303292

[20] Polis CB, Curtis KM, Hannaford PC, Phillips SJ, Chipato T, Kiarie JN, et al An updated systematic review of epidemiological evidence on hormonal contraceptive methods and HIV acquisition in women. AIDS. 2016;30(17):2665–83. Epub 2016/10/27. 10.1097/QAD.0000000000001228 27500670 PMC5106090

[21] Ralph LJ, McCoy SI, Shiu K, Padian NS. Hormonal contraceptive use and women's risk of HIV acquisition: a meta-analysis of observational studies. Lancet Infect Dis. 2015;15(2):181–9. Epub 2015/01/13. 10.1016/S1473-3099(14)71052-7 25578825 PMC4526270

[22] Ahmed K, Baeten JM, Beksinska M, Bekker L-G, Bukusi EA, Donnell D, et al HIV incidence among women using intramuscular depot medroxyprogesterone acetate, a copper intrauterine device, or a levonorgestrel implant for contraception: a randomised, multicentre, open-label trial. The Lancet. 2019. 10.1016/S0140-6736(19)31288-7 PMC6675739 31204114

[23] Hapgood H. Is the injectable contraceptive Depo-medroxyprogesterone acetate (DMPA-IM) associated with an increased risk for HIV acquisition? The jury is still out. AIDS Res Hum Retroviruses. 2019 Epub 2019/12/05. 10.1089/AID.2019.0228 . 31797677 PMC7232639

[24] Golob JL, Margolis E, Hoffman NG, Fredricks DN. Evaluating the accuracy of amplicon-based microbiome computational pipelines on simulated human gut microbial communities. BMC Bioinformatics. 2017;18(1):283 Epub 2017/06/01. 10.1186/s12859-017-1690-0 28558684 PMC5450146

[25] Passmore J, Williams B. Role of vaginal microbiota in genital inflammation and enhancing HIV transmission. International AIDS Conference; Durban, South Africa2016.

[26] Srinivasan S, Richardson BA, Wallis J, Fiedler TL, Dezzutti CS, Chirenje MZ, et al Vaginal Microbiota and HIV Acquisition Risk Among African Women. CROI; Boston, MA, USA 2018.

[27] Achilles SL, Austin MN, Meyn LA, Mhlanga F, Chirenje ZM, Hillier SL. Impact of contraceptive initiation on vaginal microbiota. Am J Obstet Gynecol. 2018;218(6):622 e1– e10. Epub 2018/03/06. 10.1016/j.ajog.2018.02.017 29505773 PMC5990849

[28] Birse KD, Romas LM, Guthrie BL, Nilsson P, Bosire R, Kiarie J, et al Genital Injury Signatures and Microbiome Alterations Associated With Depot Medroxyprogesterone Acetate Usage and Intravaginal Drying Practices. J Infect Dis. 2017;215(4):590–8. Epub 2016/12/25. 10.1093/infdis/jiw590 28011908 PMC5388302

[29] Brooks JP, Edwards DJ, Blithe DL, Fettweis JM, Serrano MG, Sheth NU, et al Effects of combined oral contraceptives, depot medroxyprogesterone acetate and the levonorgestrel-releasing intrauterine system on the vaginal microbiome. Contraception. 2017;95(4):405–13. Epub 2016/12/04. 10.1016/j.contraception.2016.11.006 27913230 PMC5376524

[30] Jespers V, Kyongo J, Joseph S, Hardy L, Cools P, Crucitti T, et al A longitudinal analysis of the vaginal microbiota and vaginal immune mediators in women from sub-Saharan Africa. Sci Rep. 2017;7(1):11974 Epub 2017/09/22. 10.1038/s41598-017-12198-6 28931859 PMC5607244

[31] Roxby AC, Fredricks DN, Odem-Davis K, Asbjornsdottir K, Masese L, Fiedler TL, et al Changes in Vaginal Microbiota and Immune Mediators in HIV-1-Seronegative Kenyan Women Initiating Depot Medroxyprogesterone Acetate. J Acquir Immune Defic Syndr. 2016;71(4):359–66. Epub 2016/02/26. 10.1097/QAI.0000000000000866 26914908 PMC4770856

[32] Yang L, Hao Y, Hu J, Kelly D, Li H, Brown S, et al Differential effects of depot medroxyprogesterone acetate administration on vaginal microbiome in Hispanic White and Black women. Emerg Microbes Infect. 2019;8(1):197–210. Epub 2019/03/15. 10.1080/22221751.2018.1563458 30866773 PMC6455113

[33] Chaban B, Links MG, Jayaprakash TP, Wagner EC, Bourque DK, Lohn Z, et al Characterization of the vaginal microbiota of healthy Canadian women through the menstrual cycle. Microbiome. 2014;2:23 Epub 2014/07/24. 10.1186/2049-2618-2-23 25053998 PMC4106219

[34] Nanda K, Callahan R, Taylor D, Wang M, Agot K, Jenkins D, et al Medroxyprogesterone acetate levels among Kenyan women using depot medroxyprogesterone acetate in the FEM-PrEP trial. Contraception. 2016;94(1):40–7. Epub 2016/03/15. 10.1016/j.contraception.2016.03.003 26972780 PMC4894753

[35] Mishell DR Jr. Pharmacokinetics of depot medroxyprogesterone acetate contraception. J Reprod Med. 1996;41(5 Suppl):381–90. Epub 1996/05/01. . 8725700

[36] Nugent RP, Krohn MA, Hillier SL. Reliability of diagnosing bacterial vaginosis is improved by a standardized method of gram stain interpretation. J Clin Microbiol. 1991;29(2):297–301. Epub 1991/02/01. 1706728 10.1128/jcm.29.2.297-301.1991 PMC269757

[37] Fredricks DN, Fiedler TL, Thomas KK, Mitchell CM, Marrazzo JM. Changes in vaginal bacterial concentrations with intravaginal metronidazole therapy for bacterial vaginosis as assessed by quantitative PCR. J Clin Microbiol. 2009;47(3):721–6. Epub 2009/01/16. 10.1128/JCM.01384-08 19144794 PMC2650913

[38] Srinivasan S, Morgan MT, Fiedler TL, Djukovic D, Hoffman NG, Raftery D, et al Metabolic signatures of bacterial vaginosis. MBio. 2015;6(2). Epub 2015/04/16. 10.1128/mBio.00204-15 25873373 PMC4453549

[39] Fredricks DN, Fiedler TL, Thomas KK, Oakley BB, Marrazzo JM. Targeted PCR for detection of vaginal bacteria associated with bacterial vaginosis. J Clin Microbiol. 2007;45(10):3270–6. Epub 2007/08/10. 10.1128/JCM.01272-07 17687006 PMC2045326

[40] Khot PD, Ko DL, Hackman RC, Fredricks DN. Development and optimization of quantitative PCR for the diagnosis of invasive aspergillosis with bronchoalveolar lavage fluid. BMC Infect Dis. 2008;8:73 Epub 2008/05/31. 10.1186/1471-2334-8-73 18510764 PMC2440748

[41] Srinivasan S, Hoffman NG, Morgan MT, Matsen FA, Fiedler TL, Hall RW, et al Bacterial communities in women with bacterial vaginosis: high resolution phylogenetic analyses reveal relationships of microbiota to clinical criteria. PLoS One. 2012;7(6):e37818 Epub 2012/06/22. 10.1371/journal.pone.0037818 22719852 PMC3377712

[42] Austin PC, Steyerberg EW. The number of subjects per variable required in linear regression analyses. J Clin Epidemiol. 2015;68(6):627–36. Epub 2015/02/24. 10.1016/j.jclinepi.2014.12.014 . 25704724

[43] Doyle R, Gondwe A, Fan YM, Maleta K, Ashorn P, Klein N, et al A Lactobacillus-Deficient Vaginal Microbiota Dominates Postpartum Women in Rural Malawi. Appl Environ Microbiol. 2018;84(6). Epub 2018/01/07. 10.1128/AEM.02150-17 29305501 PMC5835753

[44] MacIntyre DA, Chandiramani M, Lee YS, Kindinger L, Smith A, Angelopoulos N, et al The vaginal microbiome during pregnancy and the postpartum period in a European population. Sci Rep. 2015;5:8988 Epub 2015/03/12. 10.1038/srep08988 25758319 PMC4355684

[45] Thomson KA, Hughes J, Baeten JM, John-Stewart G, Celum C, Cohen CR, et al Increased Risk of HIV Acquisition Among Women Throughout Pregnancy and During the Postpartum Period: A Prospective Per-Coital-Act Analysis Among Women With HIV-Infected Partners. J Infect Dis. 2018;218(1):16–25. Epub 2018/03/08. 10.1093/infdis/jiy113 29514254 PMC5989601

[46] Achilles SL, Hillier SL. The complexity of contraceptives: understanding their impact on genital immune cells and vaginal microbiota. AIDS. 2013;27 Suppl 1:S5–15. Epub 2013/10/23. 10.1097/QAD.0000000000000058 24088684 PMC4012023

[47] van de Wijgert JH, Verwijs MC, Turner AN, Morrison CS. Hormonal contraception decreases bacterial vaginosis but oral contraception may increase candidiasis: implications for HIV transmission. AIDS. 2013;27(13):2141–53. Epub 2013/05/11. 10.1097/QAD.0b013e32836290b6 . 23660575

[48] Ahmed A, Earl J, Retchless A, Hillier SL, Rabe LK, Cherpes TL, et al Comparative genomic analyses of 17 clinical isolates of Gardnerella vaginalis provide evidence of multiple genetically isolated clades consistent with subspeciation into genovars. J Bacteriol. 2012;194(15):3922–37. Epub 2012/05/23. 10.1128/JB.00056-12 22609915 PMC3416530

[49] Santiago GL, Deschaght P, El Aila N, Kiama TN, Verstraelen H, Jefferson KK, et al Gardnerella vaginalis comprises three distinct genotypes of which only two produce sialidase. Am J Obstet Gynecol. 2011;204(5):450 e1–7. Epub 2011/03/30. 10.1016/j.ajog.2010.12.061 . 21444061

[50] Schellenberg JJ, Patterson MH, Hill JE. Gardnerella vaginalis diversity and ecology in relation to vaginal symptoms. Res Microbiol. 2017;168(9–10):837–44. Epub 2017/03/28. 10.1016/j.resmic.2017.02.011 . 28341009

