# Supplementary material for: Changes in key vaginal bacteria among postpartum African women initiating intramuscular depot-medroxyprogesterone acetate
Source: PLoS One. 2020 Mar 5;15(3):e0229586. doi: 10.1371/journal.pone.0229586 (PMC7058341; doi:10.1371/journal.pone.0229586)
Supplement: S1 File — (DOCX) [file pone.0229586.s001.docx]

**Supplemental Methods**

**DNA extraction, DNA quantification, and polymerase chain reaction**

DNA was extracted from each swab using the BiOstic Bacteremia DNA Isolation Kit (Mobio, Carlsbad, CA).[1,2] Contamination from reaction buffers and/or collection swabs was assessed with sham digests from swabs without human contact. No-template water controls were included with every assay to assess contamination from Polymerase Chain Reaction (PCR) reagents. DNA samples were tested for PCR inhibitors,[3] and 16S rRNA gene copies in each sample (total bacterial load) were measured using a broad-range qPCR assay.[2]

MicroAmp® Fast Optical 96-well reaction plates (ThermoFisher Scientific, Waltham, MA, USA) were used to setup qPCR assays. TaqMan® Fast Advanced Master Mix (Life Technologies, Grand Island, NY, USA) was used and primers were added at 0.8 μM per reaction. Assays underwent 45 cycles of amplification on the StepOnePlus real-time PCR system or the QuantStudio6 real-time PCR system (Life Technologies, Grand Island, NY, USA). Plasmid standards were run in duplicate, and sensitivity and specificity of each qPCR was assessed. Two microliters of DNA were added to each qPCR mixture and values are reported as 16S rRNA gene copies/swab. Technicians processing samples were blinded to contraception type.

**References**

1. Fredricks DN, Fiedler TL, Thomas KK, Oakley BB, Marrazzo JM. Targeted PCR for Detection of Vaginal Bacteria Associated with Bacterial Vaginosis. Journal of Clinical Microbiology. 2007;45: 3270–3276. doi:10.1128/JCM.01272-07

2. Srinivasan S, Hoffman NG, Morgan MT, Matsen FA, Fiedler TL, Hall RW, et al. Bacterial Communities in Women with Bacterial Vaginosis: High Resolution Phylogenetic Analyses Reveal Relationships of Microbiota to Clinical Criteria. PLOS ONE. 2012;7: e37818. doi:10.1371/journal.pone.0037818

3. Khot PD, Ko DL, Hackman RC, Fredricks DN. Development and optimization of quantitative PCR for the diagnosis of invasive aspergillosis with bronchoalveolar lavage fluid. BMC Infectious Diseases. 2008;8. doi:10.1186/1471-2334-8-73
