# Supplementary material for: Changes in key vaginal bacteria among postpartum African women initiating intramuscular depot-medroxyprogesterone acetate
Source: PLoS One. 2020 Mar 5;15(3):e0229586. doi: 10.1371/journal.pone.0229586 (PMC7058341; doi:10.1371/journal.pone.0229586)
Supplement: S1 Table — Abbreviations: BV, bacterial vaginosis; DMPA-IM, intramuscular depot-medroxyprogesterone acetate; HC, hormonal contraception. Enrollment: visit DMPA-IM was administered; Follow-up: visit three-months post DMPA-IM. a Exact McNemar significance probability. b No switches in detectability. (DOCX) [file pone.0229586.s002.docx]

**Table S1. McNemar’s test for change in Nugent-BV category and detectability of bacterial taxa from enrollment to 3-month follow-up by contraceptive group.**

|  | **Group** | **P-value^a^** |
| --- | --- | --- |
| Nugent-BV category  (Nugent score of 4-10 vs. 0-3) | non-HC | 0.625 |
|  | DMPA-IM | 0.344 |
| *G. vaginalis* | non-HC | -- ^b^ |
|  | DMPA-IM | 0.453 |
| *Sneathia* spp. | non-HC | 0.250 |
|  | DMPA-IM | 1.000 |
| *Eggerthella* sp. Type 1 | non-HC | 1.000 |
|  | DMPA-IM | 0.500 |
| *M. hominis* | non-HC | 0.500 |
|  | DMPA-IM | 0.500 |
| *G. asaccharolytica* | non-HC | 1.000 |
|  | DMPA-IM | 0.625 |
| *Parvimonas* sp. Type 1 | non-HC | 0.250 |
|  | DMPA-IM | -- ^b^ |
| *Parvimonas* sp. Type 2 | non-HC | 1.000 |
|  | DMPA-IM | 0.500 |
| *Megasphaera* spp. 1 & 2 | non-HC | 1.000 |
|  | DMPA-IM | 1.000 |

Abbreviations: BV, bacterial vaginosis; DMPA-IM, intramuscular depot-medroxyprogesterone acetate; HC, hormonal contraception.

Enrollment: visit DMPA-IM was administered; Follow-up: visit 3-months post DMPA-IM.

^a^ Exact McNemar significance probability.

^b^ No switches in detectability.
