# Supplementary figures and images for: Changes in key vaginal bacteria among postpartum African women initiating intramuscular depot-medroxyprogesterone acetate
Source: PLoS One. 2020 Mar 5;15(3):e0229586. doi: 10.1371/journal.pone.0229586 (PMC7058341; doi:10.1371/journal.pone.0229586)

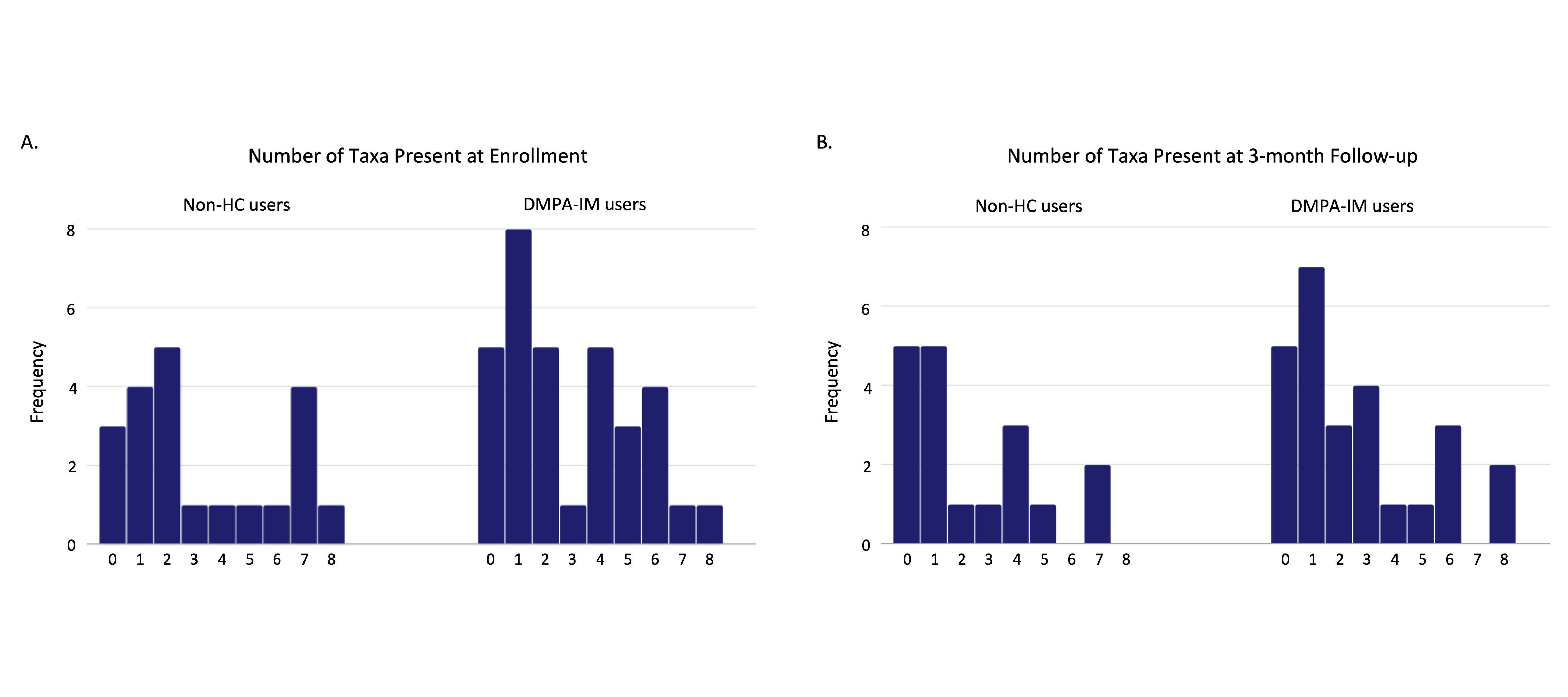

Supplement: S1 Fig — Histogram of the number of taxa present (out of the eight assessed) at (A) Enrollment (n = 54) and (B) the three-month follow-up visit (n = 44). Abbreviations: DMPA-IM, intramuscular depot-medroxyprogesterone acetate; HC, hormonal contraception. Enrollment: visit DMPA-IM was administered; Follow-up: visit three-months post DMPA-IM. (TIF) [file pone.0229586.s003.tif]

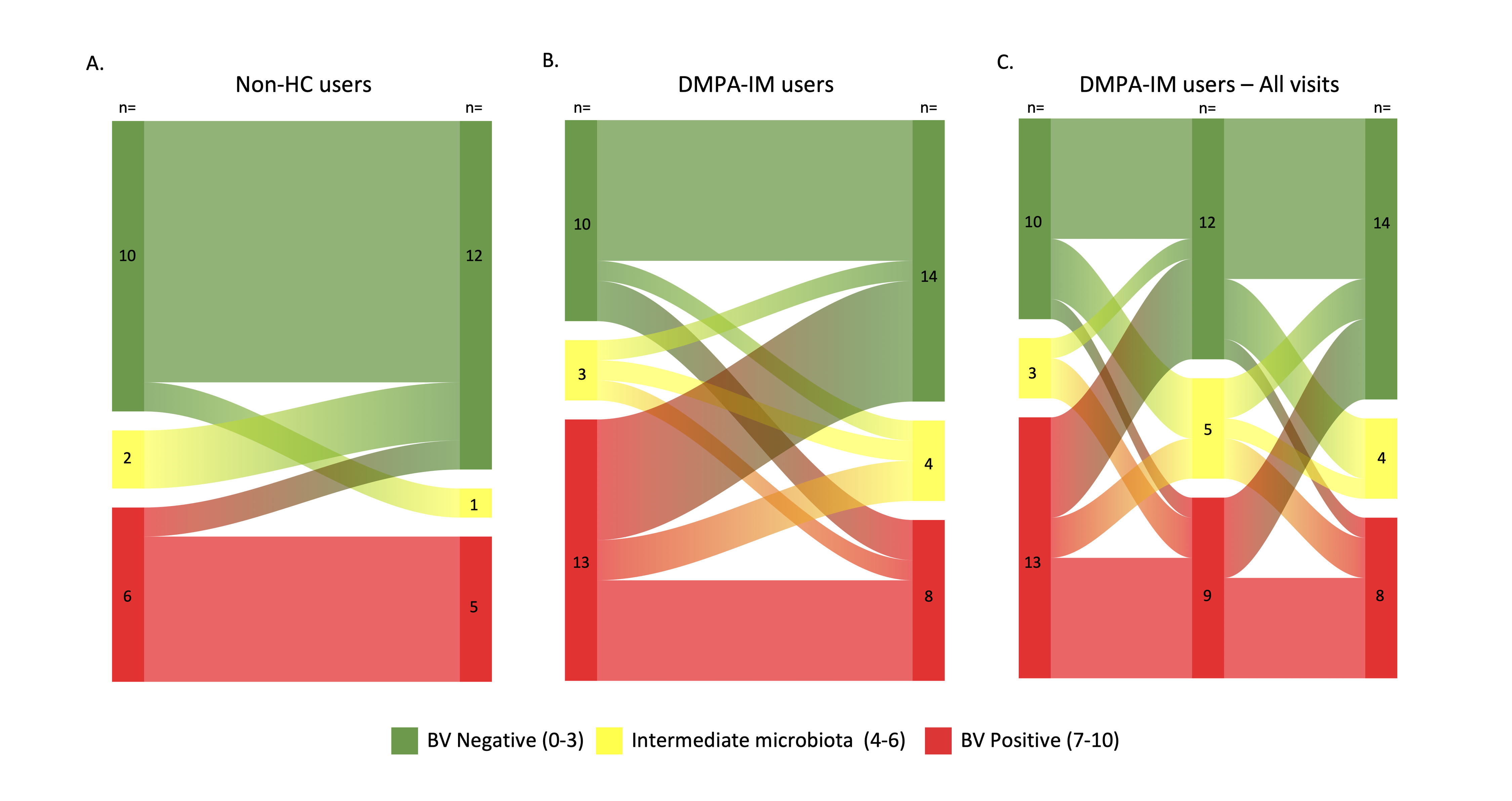

Supplement: S2 Fig — Change in Nugent category from enrollment to the three-month follow-up for (A) non-HC users (n = 18) and (B) DMPA-IM users (n = 26), among the 44 women who returned for follow-up. (C) Change in Nugent score category from enrollment to the two-week post-injection visit to the three-month follow-up visit among DMPA-IM users (n = 26) who attended every visit. Abbreviations: DMPA-IM, intramuscular depot-medroxyprogesterone acetate; HC, hormonal contraception. Enrollment: visit DMPA-IM was administered; two-week visit: visit 14 days post DMPA-IM; Follow-up: visit three-months post DMPA-IM. (TIF) [file pone.0229586.s004.tif]

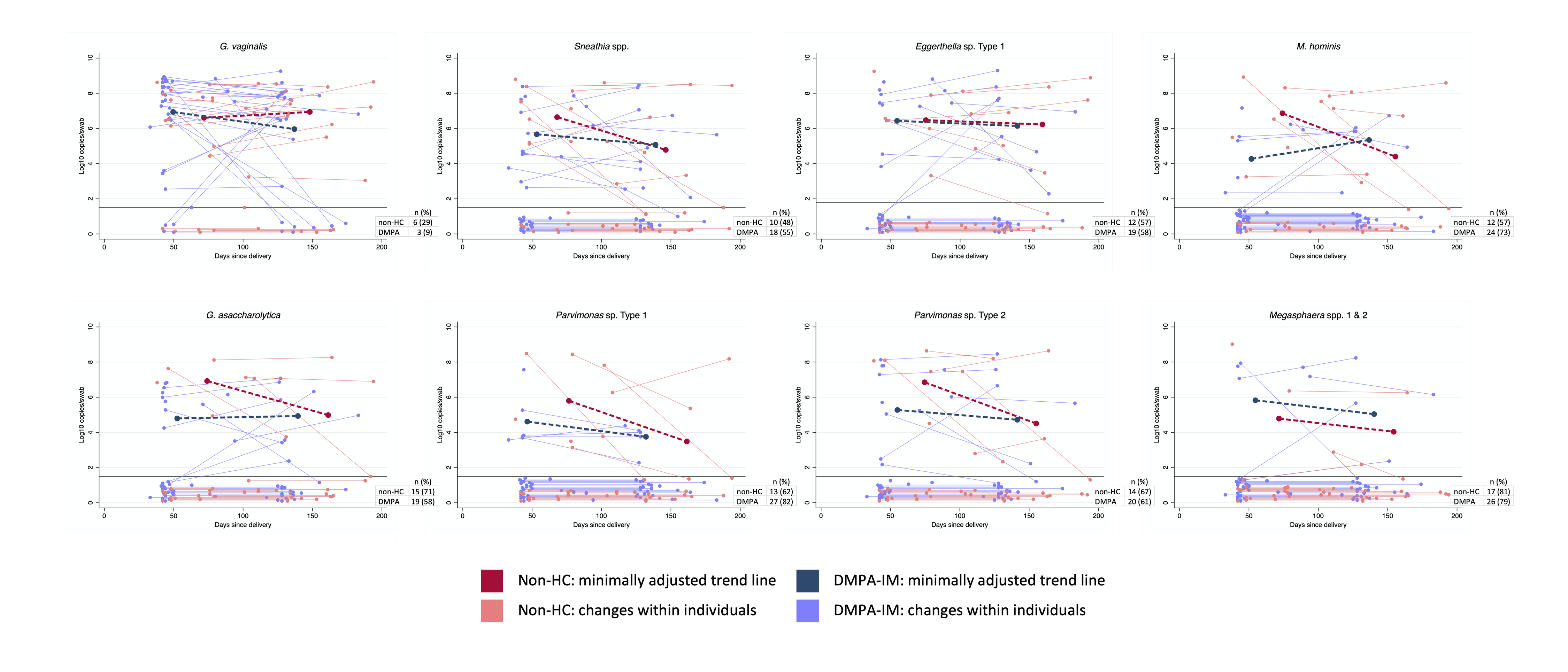

Supplement: S3 Fig — (A) Nugent score, (B) total bacterial load, and (C) concentration of vaginal taxa over time, by contraceptive group, with fitted trend lines for mean change among women ≥1 detectable value during follow-up. Abbreviations: DMPA-IM, intramuscular depot-medroxyprogesterone acetate; HC, hormonal contraception. Bacterial concentrations were log10 transformed to normalize their distribution. All values below LLD (black horizontal bar on graph) were equal, but values were jittered to allow for visualization of all observations. Trend lines for mean change in concentration among women ≥1 detectable value during follow-up within each contraceptive group were estimated using GEE with an interaction term between contraceptive group and days from delivery to enrollment and adjusted for days from enrollment to vaginal swab collection; trend lines show mean enrollment and exit dates for each group. (TIF) [file pone.0229586.s005.tif]

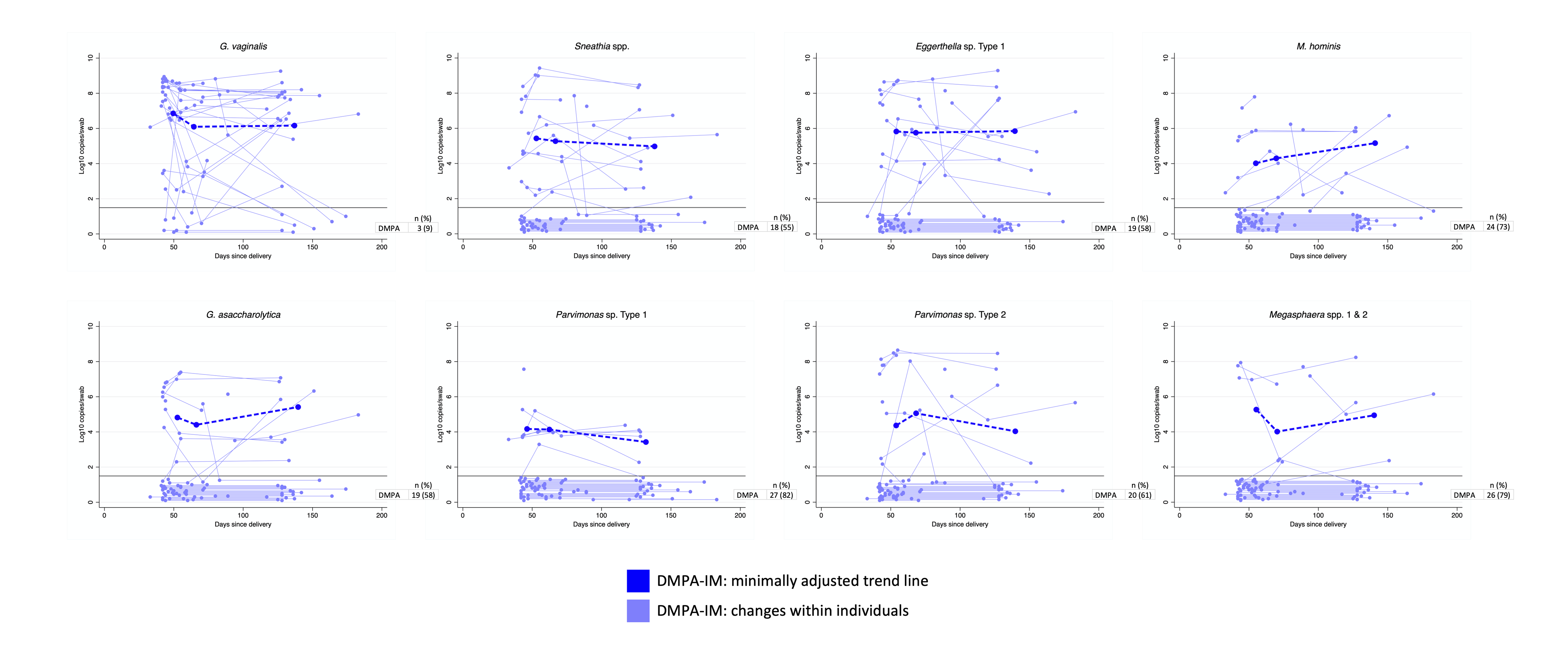

Supplement: S4 Fig — (A) Nugent score, (B) total bacterial load, and (C) concentration of vaginal taxa over time among DMPA-IM users only with fitted trend lines for mean change among women ≥1 detectable value during follow-up. Abbreviations: DMPA-IM, intramuscular depot-medroxyprogesterone acetate. Bacterial concentrations were log10 transformed to normalize their distribution. All values below LLD (black horizontal bar on graph) were equal, but values were jittered to allow for visualization of all observations. Trend lines for mean change in concentration among women ≥1 detectable value during follow-up were estimated using GEE adjusted for days from delivery to enrollment and days from enrollment to vaginal swab collection; trend lines show mean enrollment and exit dates. (TIF) [file pone.0229586.s006.tif]
